# Mannose Ligands for Mannose Receptor Targeting

**DOI:** 10.3390/ijms25031370

**Published:** 2024-01-23

**Authors:** Marija Paurević, Martina Šrajer Gajdošik, Rosana Ribić

**Affiliations:** 1 Department of Chemistry, Josip Juraj Strossmayer University of Osijek, Cara Hadrijana 8/A, HR-31000 Osijek, Croatia; marija.paurevic@kemija.unios.hr (M.P.); msgajdosik@kemija.unios.hr (M.Š.G.); 2 Department of Nursing, University Center Varaždin, University North, Jurja Križanića 31b, HR-42000 Varaždin, Croatia

**Keywords:** mannose, mannose receptor, ligand–receptor interaction, targeted drug delivery

## Abstract

The mannose receptor (MR, CD 206) is an endocytic receptor primarily expressed by macrophages and dendritic cells, which plays a critical role in both endocytosis and antigen processing and presentation. MR carbohydrate recognition domains (CRDs) exhibit a high binding affinity for branched and linear oligosaccharides. Furthermore, multivalent mannose presentation on the various templates like peptides, proteins, polymers, micelles, and dendrimers was proven to be a valuable approach for the selective and efficient delivery of various therapeutically active agents to MR. This review provides a detailed account of the most relevant and recent aspects of the synthesis and application of mannosylated bioactive formulations for MR-mediated delivery in treatments of cancer and other infectious diseases. It further highlights recent findings related to the necessary structural features of the mannose-containing ligands for successful binding to the MR.

## 1. Introduction

The mannose receptor (MR) is a transmembrane C-type lectin receptor predominantly expressed on macrophages, immature dendritic cells (DCs), and endothelial cells [1]. It acts as a pattern recognition receptor that selectively binds and internalizes various glycosylated ligands and pathogens and, thus, has an important role as a target for diverse therapeutic effects. Structurally, the MR consists of a short cytosolic region, a transmembrane region, and an extracellular cytoplasmic tail. The extracellular region contains an N-terminal cysteine-rich (CR) domain, a fibronectin (FN) type II domain, and eight C-type lectin-like domains (CTLDs), which are responsible for binding glycostructures with terminal mannose, fucose, or a *N*-acetylglucosamine (GlyNAc) portion in a calcium-dependent mode [2]. CTLDs 4 and 5 are responsible for most of the sugar-binding activity [3]. Other CTLDs are important structural units that enable higher binding affinity for the multivalent ligand recognition and geometrical flexibility to interact with different ligands [4]. Interaction of these domains with mannosylated glycoconjugates explains the binding of the MR to lysosomal enzymes, fragments of collagen, and viral glycoproteins and can also account for sensing mannose structures on the surface of various microorganisms [1,5,6,7]. Structural studies indicated that the MR, and specifically CTLD4, interacts with mannose-rich glycans through the preferential binding of the Manα1-2Man pattern [7]. Recently, Feinberg et al. premiered a valuable structural insight into defining the binding selectivity of CRD4 and the molecular mechanisms by which it is achieved using binding competition experiments, glycan array analysis, and X-ray crystallography [7].

The affinity of sugar-binding pockets for a single-monomannoside ligand is low but can be enhanced by the multivalency [8], especially by means of multivalent presentation on larger ligands on a polyvalent core or carrier systems such as liposomes, nanoparticles, or dendrimers. The mannosylation of peptides and antigens is a notoriously powerful strategy for enhancing antigen presentation and T-cell activation. Therefore, it is an important method for the development of new vaccines against different pathogens and therapeutic cancer vaccines [9,10,11]. Most of the earlier strategies involved the use of natural mannose conjugates, while recently, synthetic ligands with high affinity and specificity for MR were developed [12,13,14,15]. In addition, some high mannose glycans can act as antigens and/or adjuvants [16,17]. For example, the conjugation of a mannose-based copolymer, synthetic glycol-adjuvant p(Man-TLR7), to protein antigens resulted in higher humoral and cellular immunity in mice models when compared to antigens lacking mannose targeting or TLR7 ligand [18]. Lately, many efforts have been devoted to the synthesis of multivalent mannosystems, such as dendrimers, micelles, and liposomes. Multiple functionalizations of the carrier systems with a mannose pendant were proven to be a valuable approach for fast, selective, and efficient delivery of imaging and therapeutic agents to macrophages [19,20,21,22]. Furthermore, it was shown that branching, multimerization, and the number of mannose moieties provide MR-based uptake and, thus, macrophage activation [23,24]. In more detail, the arrangement and location of the mannose units, such as the distance between the mannose groups (shorter spaces between mannose units), are important for effective receptor targeting and binding [24]. MR has a higher affinity for branched mannosylated ligands than for linear structures [12,25]. Also, the linkage between the carrier and the mannose derivative determines stability under physiological conditions and, thus, the efficiency of the system. Conventionally, the coupling procedure is commonly initiated by the amine-carboxylic acid reaction, and lately, copper-catalyzed click reactions have emerged due to their orthogonality, specificity, speed, and efficiency [26,27]. This review focuses on the recent development of different mannosylated ligands with additional therapeutic functionalization specifically designed for MR targeting. Moreover, this work presents the benefit of mannosylation as an efficient strategy to improve the uptake and targeting ability of these systems towards the MR.

## 2. Structure and Function of MR

The MR, also known as CD206, belongs to a group of multilectin receptor proteins containing multiple C-type lectin domains (CTLDs) present within a single polypeptide backbone [28,29]. C-type lectins are Ca^2+^-dependent glycan-binding proteins with carbohydrate-recognition domains (CRDs) [30]. The MR is primarily expressed by macrophages and dendritic cells and has an important role in endocytosis as well as antigen processing and presentation. Due to its ability to recognize, bind, and internalize a broad range of endogenous and exogenous ligands, it plays a significant part in host immune responses, including both innate and adaptive immunity [1,28,31,32,33,34]. MR CTLDs are responsible for the recognition and binding of glycoconjugates (mannans, glucans, lipophosphoglycans, etc.) with terminal mannose, fucose, or *N*-acetylglucosamine (GlcNAc) in a Ca^2+^-dependent manner [1,7,28].

The MR was first identified on rabbit alveolar macrophages as a receptor involved in the clearance of endogenous glycoproteins [32,35]. Later, it was shown that MR is expressed not only by tissue macrophages but also by dendritic cells and various hepatic and lymphatic endothelial cells [2,5,32,36]. The MR expression levels are closely correlated with the activation status of MR-expressing cells and can be distinctively regulated by cytokines, prostaglandins, transcription factors, etc. The increase in MR expression levels was observed in response to cytokines interleukin-4, (IL-4), IL 10, and IL-13, whereas the interferon-γ exerted a negative effect [1,32,37].

**Figure 1:**
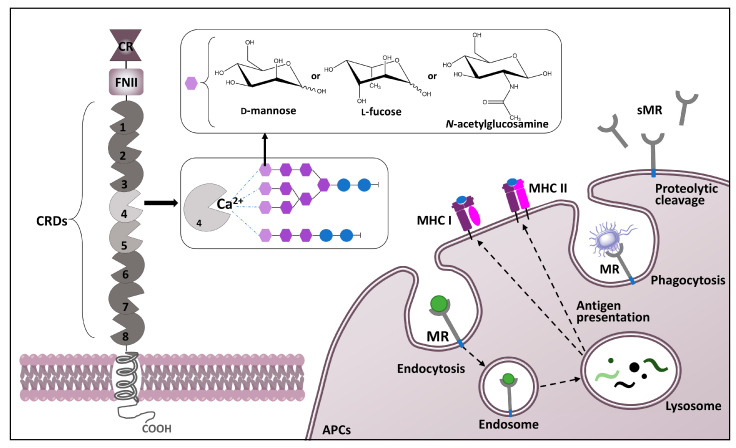
Structure of the mannose receptor (MR) and the overview of its cellular functions. MR consists of distinct functional domains, including the cysteine-rich (CR) domain, which specifically recognizes sulfated carbohydrates; fibronectin type II (FNII) domain with an affinity for various types of collagen; eight carbohydrate recognition domains (CRDs), binding to complex carbohydrate ligands; one transmembrane domain and the C-terminal internalization motif, implicated in the process of transferring ligands into the cell. CRD4 exhibits binding affinity to linear and multibranched carbohydrate ligands terminating in mannose, fucose, or *N*-acetylglucosamine in calcium-dependent manner. MR is both an endocytic and a phagocytic receptor involved in the phagocytosis of various pathogens. In endosomal pathway, attachment of glycosylated ligands to MR results in their internalization, followed by the degradation in the lysosome. Degraded ligands can be processed for the presentation to the surface of antigen-presenting cells (APCs) through major histocompatibility complexes I and II (MHC I and MHC II). The soluble form of MR (sMR) can be released in the extracellular space due to the proteolytic cleavage by metalloproteases.

MR is a 175 kDa type I transmembrane protein consisting of five domains (Figure 1): one transmembrane domain, a short cytosolic domain that has a main function in receptor internationalization and recycling [28,35,38], and the extracellular region consisting of an N-terminal cysteine-rich (CR) domain, fibronectin II (FNII) repeat region, and eight C-type lectin-like carbohydrate recognition domains (CTLDs) [28,31]. The N-terminal CR domain has the capacity to bind glycoproteins with sulfated sugars that terminate in 4-SO_4_GalNAc, including sulfated hormones lutropin and thyroid stimulating hormone. The CR domain can also bind other classes of sulfated oligosaccharide ligands like chondroitin sulfate A and B and 3-sulfated oligosaccharides of Lewis^a^ and Lewis^X^ types terminating in 3-SO_4_Gal [1,28,39]. The FN type II domain follows the CR domain and has a role in the binding of collagen, in particular collagen types I, II, III, and IV [35,40].

The domain organization of the MR is described above, and as already mentioned, the extracellular part of MR consists of eight CTDLs. CTLDs play a key role in the binding of MR to sugars terminated in mannose, fucose, or *N*-acetylglucosamine. The structure of CTLDs is characterized by a fold containing two alpha helices and two small antiparallel beta sheets stabilized by covalent and non-covalent interactions, including nested disulfide bonds [41]. Hydrophobic residues contribute to the stabilization of the fold, forming a hydrophobic core. In functional CTLDs, sugar interactions take place in the hydrophobic fold, which facilitates the coordination of Ca^2+^ ions and sugar residues [41,42]. Studies on the MR CTLD sequences show that, although CTLDs 1-8 have the essential residues for the formation of the hydrophobic fold, only CTLD4 and CTLD5 possess the necessary residues for Ca^2+^-dependent sugar ligation [28,32].

Since CTLDs 4 and 5 are responsible for most of the sugar-binding activity, they are, therefore, commonly called carbohydrate recognition domains (CRDs) 4 and 5. CTLD4 is the only CTLD that binds larger carbohydrates in the absence of other domains [4]. Different techniques were employed for the detection and quantification of carbohydrate-MR interactions. Surface plasmon resonance (SPR) assays, ELISA (enzyme-linked immuno-sorbent assay), NMR titration, isothermal titration calorimetry (ITC), electron microscopy, X-ray crystallography, and molecular modeling methods are some of the most used. Recently, Feinberg et al. presented valuable structural insight into defining the binding selectivity of CRD4 and the molecular mechanisms by which it is achieved using binding competition experiments, glycan array analysis, and X-ray crystallography [7].

CRD4 binds sugar ligands mediated through Ca^2+^ coordination in their primary carbohydrate-binding sites. Conserved Ca^2+^ without bound ligand is surrounded by five amino acid residues: two glutamic acids (Glu725 and Glu733), two asparagines (Asn727 and Asn747), and one aspartic acid (Asp748). Additionally, Tyr729 contributes to the orientation of mannose in the primary binding site by forming van der Waals contact with carbohydrates. Interestingly, high-resolution crystal complexes demonstrate that the Ca^2+^-coordinated by 3- and 4-OH groups of D-mannose can interact in alternate orientations with the same glycan/monosaccharide [7].

The binding affinity of MR for glycosyl groups is determined by the terminal sugar moieties. The MR exhibits a notable preference for L-fucose, followed by mannose, GlcNAc, glucose, and D-xylose, in descending order of affinity [29,43]. Additionally, the glycosidic linkages play a role in influencing binding, with α1-6 mannan displaying a higher affinity for the mannose receptor compared to α1-2 or α1-3 mannan. Comparative experiments involving branched and linear oligosaccharides indicate a pronounced preference for branched glycosyl groups, while the presence of two terminal mannoses results in high-affinity binding [44]. Furthermore, a methyl modification of mannose enhances affinity five-fold, suggesting that hydrophobic interactions contribute to the mechanism of recognition [29,44].

The conformation upon ligand binding and the release of MR are regulated by environmental pH. Structural studies revealed that MR adopts extended and flexible conformation in physiological or basic pH and a more condensed conformation in acidic environments [45,46,47]. Additional structural studies of conformational changes upon ligand binding, along with comprehensive biophysical and in silico characterization of binding interactions, would improve the understanding of the mechanisms of MR and relieve the development of therapeutic strategies against related diseases.

Extensive examination of the endocytic properties of MR revealed that the internalization of the receptor occurs even without ligand binding. In normal conditions, MR constantly recycles between the plasma membrane and early endosome, with around 70% of the receptor being localized inside the cell [1,35]. The internalization of MR and its deliverance into the endosomal system occurs via clathrin-coated vesicles. MR poses distinct endocytosis-associated motifs in the cytoplasmic domain: a motif based on a conserved tyrosine residue and a dihydrophobic motif [37,38].

Membrane-bound MR can exist in monomeric and multimeric states [48]. Also, metalloproteases can proteolytically cleave the extracellular region of MR directly after the transmembrane region, releasing a soluble form of MR (sMR) in extracellular space [1,49,50]. The presence of sMR was detected in the supernatant of cells expressing MR, in the serum of mice and humans, and, recently, in extracellular vesicles [1,49,50,51,52]. The elevated levels of sMR were observed in various inflammatory diseases and were positively correlated with disease severity and mortality. Thus, sMR was suggested as a novel inflammation biomarker [1,53].

The involvement of MR in innate immune responses was recognized early. The innate immune system presents a first-line defense against pathogens, mainly through pattern recognition receptors (PPRs) present on macrophages and dendritic cells (DC) [54]. When attached to a ligand present on the surface of an invading microorganism, the PRRs can facilitate their uptake through phagocytosis and/or initiate intracellular signals, ultimately triggering an activation of the host cell [29,54]. The MR acts as PRR via its CTLDs binding mannose and *N*-acetylglucosamine, which are often found as terminal residues on surface glycoproteins of many microorganisms [28,55]. It was shown that MR recognizes and binds a variety of bacteria [56,57,58], viruses [59,60], fungi [61,62], and parasites, determining them for endocytosis by macrophages and subsequent degradation in lysosomes [28].

However, MR serves functions beyond the mere phagocytosis of pathogens. It exhibits multiple intracellular responses upon binding to microorganisms, including lysosomal enzyme secretion [63], cytokine secretion, and the modulation of other cell surface receptors [64,65]. Additionally, glycosylated viral envelope proteins, such as those found in HIV and HSV, induce IFN-α production by dendritic cells [65]. This response can be hindered by sugars specific to the MR, suggesting its crucial role in recognizing enveloped viruses by DCs [65].

As one of the key receptors in the host defense mechanism, MR also has a role in adaptive immunity. Being antigen-specific, adaptive immunity enables long-term recognition and delivers an improved immune response upon subsequent interactions with the pathogen [29] and has the ability to generate a range of T- and B-cells and antigen-specific receptors, including major histocompatibility complexes I and II (MHC I and II). Immature DCs have a crucial role in antigen processing and presentation to T-cell receptors after recognizing and internalizing pathogens. It was shown that MR, present on the DCs, causes their maturation upon the binding of glycosylated ligands [12,66,67]. DCs utilize endocytosis through MR, starting in small coated vesicles. Following internalization, both the MR and its ligand transition to larger vesicles, ultimately co-localizing with MHC class II molecules in lysosomes. After processing, antigens are loaded onto MHC II molecules and presented to T-cells. Notably, mannosylated peptides and proteins demonstrate a significantly higher efficiency (200–10,000-fold) in stimulating T cells compared to their non-mannosylated counterparts [12,31]. Furthermore, the MR-mediated uptake of mannosylated antigens by immature DC results in a 100-fold enhanced presentation of soluble antigens to T-cells compared to antigens internalized via the fluid phase [67].

Since MR plays an important role in immune homeostasis and the uptake and processing of foreign antigens during various pathological conditions, it was recognized as a potential target for imaging, diagnosis, and therapy [1,5,12]. MR can be used to deliver drugs, e.g., mannose-conjugated antibiotics, to macrophages infected by intracellular bacteria [68]. The functionalization of different carriers with mannose or anti-MR nanobodies was utilized for targeting MR, including cell-specific gene delivery systems [69]. MR was the subject of investigations involving MR-mediated gene transfer into macrophages, utilizing mannosylated cationic liposomes that demonstrated high transfection activity owing to MR recognition. Extending from this, several studies provided evidence supporting the targeting of the MR pathway for vaccine delivery. Notably, a novel DNA vaccine formulation was shown to enhance cytotoxic T-lymphocyte activity through efficient gene delivery to dendritic cells (DCs) via MR-mediated endocytosis. Furthermore, the MR endocytic pathway serves as a viable means to deliver DNA-based vaccines into antigen-presenting cells using mannosylated liposomes.

Tissue macrophages are crucial cells of the innate immune system and integral communicators for the establishment and sustenance of the adaptive immune system. Their phenotype and functions can greatly vary depending on the originating tissue and the activation state. According to their phenotype and function, macrophages are divided into M1 and M2 macrophages. M1 macrophages mainly mediate pro-inflammatory processes, while M2 macrophages possess anti-inflammatory activity. Also, tumor-associated macrophages (TAM) are M2-type macrophages with high MR expression [70,71]. Their role in tumor proliferation invasion was described, and therefore, targeting MR on TAMs to mediate MR-directed anticancer drug delivery was proposed [72,73].

## 3. Mannose-Based Glycomimetics as Ligands for MR

A large variety of multivalent platforms were employed as cores for mannose-mediated MR presentation, including liposomes, peptides, proteins, various polymers, micelles, dendrimers, dendrons, and nanoparticles. Sugars have good targeting specificity, low toxicity, and biocompatibility. With the development of glycobiology, sugar chain engineering and glycomics, the application of sugars in medicine was developed quickly [74]. These sugar structures achieve the effective and active targeting of various tissues and cells and have the ability to load different kinds of drugs, genes, and therapeutic RNA molecules [75]. Recently, an important study of trimannose-coupling for effective delivery of inhaled oligonucleotides to pulmonary macrophages was published as the first mannosylated therapeutic candidate for COVID-19 [76]. Lately, mannose is acquiring more and more interest as a promising target ligand in cancer therapy.

**Figure 2:**
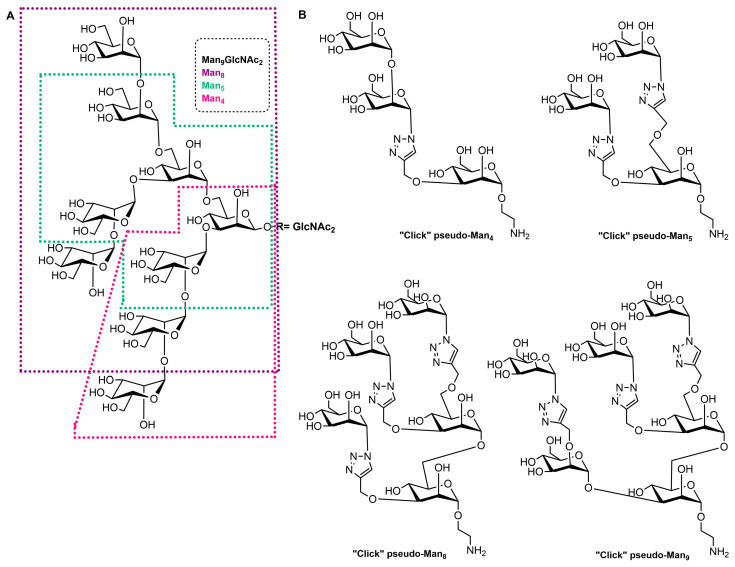
(**A**) Structure of the Man9GlcNAc2 oligosaccharide with the Man4, Man5, and Man8 substructures highlighted with appropriate colors. (**B**) Structures of the “click” pseudo-high-mannose mimics.

High-mannose structures that cover the surface of many pathogenic microorganisms are readily recognized by the MR CRDs. All complex mannose glycans share the common pentasaccharide core Manα(1-6)[Manα(1-3)]Manβ(1-4)GlcNAcβ(1-4)GlcNAc (Man_3_GlcNAc_2_) carrying on to six additional mannose residues (Figure 2A). Mannan is a polysaccharide of mannose found in the cell wall of *Saccharomyces cerevisiae* (yeast) that binds to the MR CRDs and causes an immune response involving the up-regulation of co-stimulatory molecules and pro-inflammatory cytokines [12]. Due to this ability, some vaccine design attempts that mimic yeast mannose cell walls were designed. In addition, studies with synthetic mucins and some other synthetic glycopeptides that mimic natural oligomannosides were carried out in order to investigate how mannose spacing and valency affect the lectin recognition and mode of binding [77,78]. In addition, a family of branched mannooligosaccharide mimics of natural compounds, prepared by exploiting copper(I)-catalyzed azide-alkyne cycloaddition ligation strategy, was demonstrated, and their binding affinities towards the human macrophage MR (rhMMR) was examined by François-Heude and co-workers [79]. The authors replaced the demanding internal mannopyranosyl units with triazole rings while retaining the terminal mannose display (Figure 2B). The binding potency of the new pseudo-Man derivatives to concanavalin A and rhMMR was comparable to that of their respective natural partners [79,80].

For the past four decades, several other groups reported the synthesis of such structures; anyway, the preparation of high-mannose structures remains very challenging, as well as efficient incorporation into delivery systems. In general, the synthesis of multivalent mannosylated conjugates requires the attachment of mannose or its appropriate derivative (natural or synthetic ones) on a functionalized scaffold. Mono-, di-, oligo-mannose, or a polymer of mannose (mannan) are usually used in mannosylation reactions. Conventionally, the coupling procedure is commonly initiated by the amine-activated carboxylic acid reaction, copper-catalyzed click reactions, and, lately, the copper-free click-based strategy [13,18,26,27,79,80]. Apart from covalent synthetic strategies, the coupling of a ligand to a nanoparticle can be accomplished by electrostatic interactions [81,82].

Among non-covalent nanoparticle bioconjugation strategies, the spontaneous self-assembly encapsulation or controlled nanoformulation of components, including nanoprecipitation or in situ hydrogel formation, is an increasingly applicable technique for the preparation of this type of functionalized carrier. Self-assembled delivery systems such as micelles, liposomes, and lipid nanoparticles are increasingly interesting due to their biocompatibility, simple preparation, encapsulation of both hydrophilic and hydrophobic cargoes, and some other advantages [83]. The non-covalent fusion or coating of previously prepared nanoparticles with targeting ligands, commonly by electrostatic interactions, is also one of the well-known methods of preparing functional nanoparticles [84].

### 3.1. Mannosylated Peptides and Proteins

Mannosylated antigens are one of the most effective ligands for targeting the MR and obtaining high-quality immune responses. Common mannosylated vaccine antigen candidates include peptides and proteins. Chemically explained, glycosylation is used to increase peptide solubility and oral bioavailability to obtain a broader reactivity and to provide more conformational properties to synthetic peptides [85]. Biologically, most molecules involved in the immune system (cellular receptors, cytokines, and antibodies) are glycosylated due to their assembly, stability, cell surface exposure, or secretion and recognition [86]. Additionally, glycosylation increases the specificity of peptide vaccine candidates towards the desired cellular target.

**Figure 3:**
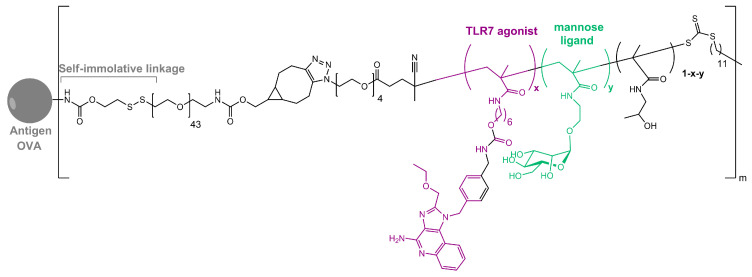
Structure of OVA-p(Man-TLR7).

As in nature, synthetic glycosylated proteins and peptides with an MR-tagging ability occur as both *O*- or *N*-linked glycol moieties. An earlier study by Gustafson et al. demonstrated MR’s high affinity for both *N*- and *O*-linked mannosylated mucin-type fusion proteins [87]. In addition, the mucin-type fusion ovalbumin (OVA) protein carrying multiple oligomannose structures enhances antigen-specific antibody and T lymphocyte responses, thus affecting the humoral and cellular anti-OVA responses in a mice model. This type of protein has great potential to work as a universal antigen-presenting cell (APC)-targeting molecule since it was shown that it can successfully target the carbohydrate recognition domains of MR, MBL, and DC-SIGN receptors [87,88]. Some of our research showed that the mannosylation of previously prepared adamantane-containing desmuramyl peptides resulted in the amplification of its immunostimulating activity in an in vivo mouse model [89,90,91]. Recently, the conjugation of the mannose-based copolymer, synthetic glycol-adjuvant p(Man-TLR7), and OVA resulted in higher humoral and cellular immunity in a mice model when compared to antigens lacking mannose targeting or a TLR7 ligand (Figure 3) [18]. The authors first synthesized p(Man-TLR7), which is a synthetic polymeric glycol-adjuvant composed of two functional monomers that target dendritic cells via mannose-binding receptors or activate DCs via Toll-like receptor 7, using the reversible addition–fragmentation chain transfer (RAFT) polymerization. Then, a copper-free cycloaddition reaction was used to conjugate p(Man-TLR7) to antigens. This method has potentially wide application due to the insensitivity of RAFT polymerization to a wide variety of functional groups and the simplicity of conjugation strategy, which can be used for both amine-containing peptides and whole proteins [92]. Antigen-p (Man-TLR7) conjugates generated a pronounced increase in the magnitude and quality of the humoral immune response, expanding the neutralizing antibody repertoire and antigen-specific memory B cells. Additionally, the newly prepared adjuvant localized immune stimulation to the lymphatic organs, avoiding an acute systemic inflammatory response.

**Figure 4:**
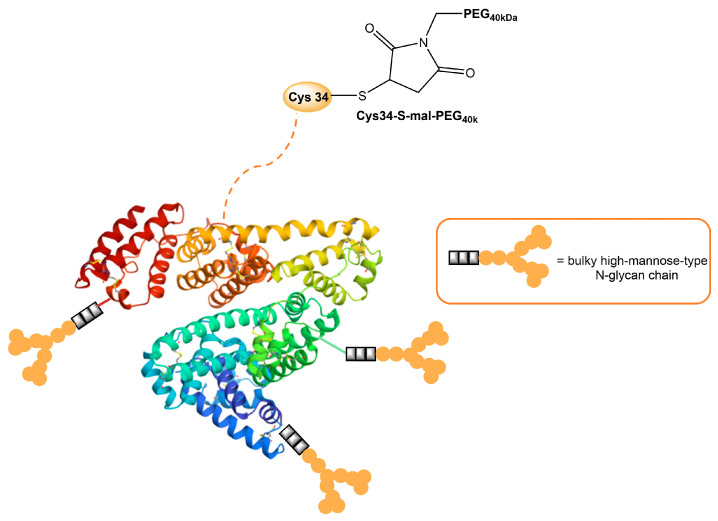
Model of HSA modified with PEG and high-mannose ligands for targeted delivery of paclitaxel to MRs on cancer stoma cells.

Human serum albumin (HSA), a non-glycosylated protein, is also an attractive carrier for drug delivery systems [93]. Chemically modified Man-albumins are largely taken up by the liver via an MR-mediated mechanism. Hirata et al. designed and genetically engineered a recombinant oligomannosylated-HSA mutant that can be selectively delivered to the liver via MR on the liver non-parenchymal cells (and, thus, work as a carrier for liver-selective therapeutics) [94]. Recently, a dual-modified albumin in which one molecule of polyethylene glycol (PEG) was conjugated to the Cys34 residue in Man-HSA (Figure 4) was developed. This trivalent system preferably enables the dual-targeting to cancer-associated fibroblast and tumor-associated macrophage-specific MRs (CD206 and CD280), which resulted in a seven times stronger suppression of melanoma growth in B16F10 tumor-bearing mice, when the effect is compared to PEGylated albumin [95]. Furthermore, the PEG-Man-HSA conjugate mediated the disruption of the tumor microenvironment by polarizing tumor macrophages toward the M1 profile.

**Figure 5:**
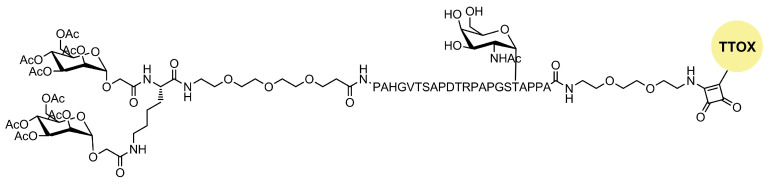
Structure of mannosylated MUC glycopeptide anticancer vaccine.

A novel tumor-associated mucin MUC1 glycopeptide anticancer vaccine functionalized with covalently linked divalent mannose ligands induced much stronger specific IgG immune responses in mice than the non-mannosylated reference vaccine. Additionally, mannose-binding led to increased numbers of macrophages, dendritic cells, and multiple T cell activation in the local lymph organs. Mannose subunits were introduced using N-terminal mannosyl glycolic acid coupled to both amino groups of a terminal lysine (Figure 5). The tetra-mannosylated conjugate was better recognized by MR and potentially presents a promising target for anti-cancer immunotherapy [15].

Pan et al. designed and synthesized a mannose-modified peptide-polymer hybrid CPP-LPEI as a gene carrier in which CPP presents cell-penetrating peptide, short cationic peptide, and LPEI less toxic low-molecular-weight polyethylenimine. The prepared hybrid served as a vector for TRAIL plasmid delivery in colorectal cancer treatment [96]. The Man-CPP-LPEI/TRAIL hybrid vector effectively induced apoptosis and the inhibition of tumor growth with minimal toxicity in vitro and undetectable toxicity in vivo in HCT116 tumor-bearing mice. The mannosylation of the conjugate was performed due to the recent discovery of a high expression of MRs on colon cancer cells and the ability of mannosylated liposomes to target colon tumors [97].

Over the last couple of decades, peptide-based hydrogels have gained increasing attention for biomedical applications and diagnostic research considering their particular properties, such as their biocompatibility, biodegradability, tuneability,hydrophilic–lipophilic balance of the structure, tissue-like elasticity, and non-cytotoxicity [98]. Considering these properties, recently, Dowari and coworkers prepared a mannose-containing composite hydrogel by combining a self-aggregating short peptide (Nap-FFGE-NH_2_) and a mannose-containing non-aggregating peptide (Nap-FF-mannosyl) [99]. Nap-FF is a small peptide hydrogelator that can form versatile biofunctional nanofibrous structures [100]. Synthesized composite hydrogel structures provided a potential candidate for the Leishmaniasis treatment via macrophage MR delivery.

Numerous nanotechnologies with advanced functional capabilities were developed [101]. A wide spectrum of these can be used as peptide delivery systems for the improvement of the efficacy of cancer vaccines, which will be discussed later in the paper [102].

### 3.2. Mannosylated Lipids and Liposomes

Liposomes are spherical and self-assembled vesicles formed in the aqueous phase from one or more phospholipid bilayers with the polar groups of phospholipid heads oriented to the inner and outer water phases. They are widely used as bioactive platforms for drug and antigen delivery due to their capability to entrap or encapsulate hydrophilic, amphipathic, and lipophilic molecules [103]. Mannosylated liposomes were constantly shown to favorably target macrophages, increasing cellular uptake, both in vitro and in vivo. Mannosylated liposomes can be prepared through the attachment of mannose onto a desired carrier through non-covalent conjugation or by covalently attaching mannose derivatives to liposomes [13]. In the past few decades, numerous strategies have evolved in order to advance a suitable liposome structure for macrophage targeting [9,24,104,105,106]. Several strategies, such as the incorporation of variable amounts and different combinations of phospholipids in the bilayer and the polymeric steric stabilization with hydrophilic PEGs, were attempted to upgrade their in vivo stability [107,108,109]. The incorporation of cholesterol in liposome formulation reduces the permeability of the lipid bilayer, increases liposome stability, and reduces the rapid release of encapsulated bioactive molecules [110]. Spacer length and flexibility between the mannose moiety and the surface of the liposome are also meaningful factors for efficient MR recognition [104,111,112,113]. In addition, the size of the vesicle is important for the circulation half-time of liposomes [103,114].

**Figure 6:**
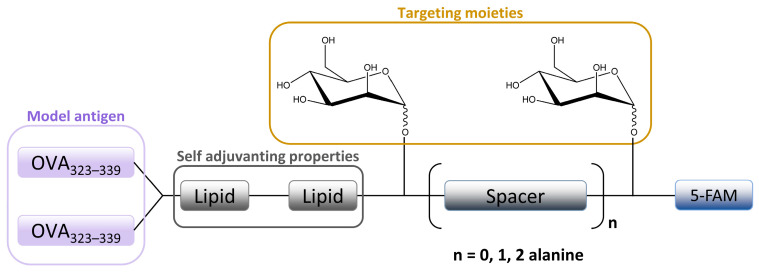
Schematic image of mannosylated lipopeptide vaccines developed by Sedaghat et al. used to target DCs and macrophages MR. Linear arrangement of two mannose units are separated by an alanine spacer attached to the ovalbumine epitope.

The in vitro MR-mediated uptake in the APC studies of Sedaghat et al. confirmed the importance of the presence of both mannosyl and lipidic moieties in lipopeptide vaccines for stronger MR binding compared to non-mannosylated compounds [24]. It is well known that the lipidation of peptides is an effective method for improving the metabolic stability and the immune-stimulatory properties of peptide-based vaccines [115,116]. When combined with mannosylation, a specific targeting ability can be achieved. A library of fluorescently labeled mannosylated lipopeptides, with the ovalbumin epitope incorporated (Figure 6), was synthesized using fluorenylmethyloxycarbonyl (Fmoc) solid phase synthesis (SPPS). This research is an extension of previous studies in which it was shown that the distance between the mannose groups plays an important role in the uptake of mannosylated compounds through the MR on monocytes [117,118]. More specifically, shorter spacers between the mannose units were shown to be more effective [9,118]. Recently, Zhang et al. designed mannose-modified liposomes, with antagonistic peptides as co-delivery systems grafted onto a liposome surface, that successfully activated macrophages and stimulated the secretion of various cytokines. The additional hyaluronic acid functionalization of liposomes improved the systemic circulation stability of complexes in vivo and promoted its accumulation in tumor sites [119].

Mannose-modified carboxylated curdlan-coated liposomes were prepared and tested for APC selectivity, cross-presentation efficiency, and antitumor effects [120]. Specific in vivo and in vitro tests showed that these liposomes are highly recognized by macrophage cell lines and APC cells in the spleen. Also, liposomes achieved the cytoplasmic delivery of cargo and promoted the cross-presentation of the model antigen, which led to the induction of strong antitumor effects in tumor-bearing mice.

Various mannosylated PEGylated lipids and liposomes were synthesized in order to improve macrophage targeting. Hagimori et al. designed novel, highly functional mannose-PEGylated liposomes with an improved in vitro MR-binding affinity using s serine-glycine repeat spacer between the mannose and lipid moieties [121]. Recently, a nanoprodrug composed of budesonide palmitate and the mannose-PEGylated lipid was prepared. In vitro studies on RAW 264.7 macrophages demonstrated the safety of prodrug administration and anti-inflammatory activity [122]. Different mannose-PEGylated and other polymer-augmented liposomes (PALs) were developed to provide improved cytosolic delivery of streptomycin to alveolar macrophages [123]. Herein, the streptomycin-loaded PALs showed significantly improved intracellular antibacterial activity in a Francisella-macrophage co-culture model when compared with free streptomycin or PEGylated streptomycin liposomes. Also, the mannose targeting capability of the PALs internalization was considerably higher compared to non-targeted PEGylated liposomes. Mannosylated PAL carrier systems designed in this way provide important features for the intracellular delivery of therapeutics with poor membrane permeability. Drug-free mannosylated liposomes and PEGylated liposomes were also recently developed, and cytotoxicity, cellular internalization, immunostimulatory activity, MR-targeting efficiency, and antitumor activity were evaluated in vitro and in vivo [124]. In the obtained results, mannosylated liposomes exhibited superior in vitro cellular internalization and tumor penetration through MR-mediated tumor-associated macrophages (TAMs). Recent studies also based on the design and application of mannosylated liposomes for the purpose of TAM-targeted delivery showed great potential in the regulation of the tumor microenvironment, as well as a promising approach for specific and non-invasive TAM-targeted imaging [125,126,127]. In addition, various PEGylated mannose–cholesterol conjugates were synthesized and used for enhanced in vitro mRNA delivery to DC cells by liposomes [113]. The uptake study revealed that prepared liposomes enhanced mRNA expression mainly through DCs MR, indicating the importance of the MR’s involvement in the mRNA vaccine delivery system.

The codelivery of mannose-modified liposomes, co-encapsulated with dihydroartemisinin and doxorubicin chemotherapeutics, for drug-resistant colon cancer therapy was described by Kang and co-workers [97]. The administration of the Man-liposomes resulted in a tumor inhibition rate of 88.6%, compared to that of 47.5% or 70.5% for the treatment with free doxorubicin or free doxorubicin with dihydroartemisinin, respectively. This combined formulation inhibited the growth of the drug-resistant colon cancer cells and, thus, can be applied for targeted delivery to cancer cells overexpressing the MR.

**Figure 7:**
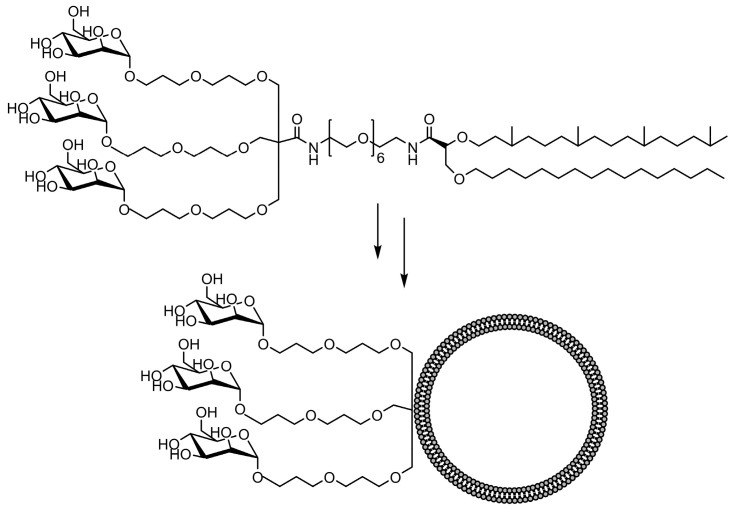
Schematic image of a tri-antenna molecule of α-D-mannopyranoside liposome, tri-Man liposome, prepared by Gao et al [128].

The comparative binding and uptake of liposomes decorated with various mannose oligosaccharides by cells expressing the MR or DC-SIGN was recently described by Gao and co-workers [128]. A series of glycolipids comprising either mannose, a tri-antenna molecule of α-D-mannopyranoside, the [Manα1-3(Manα1-6)Man], the pseudo-Man4 or pseudo-Man5 were synthesized and embedded in fluorescein-labeled liposomes. A tri-antenna α-D-mannopyranoside X showed the best potential for targeting liposomes to MR (Figure 7).

The synthesis and evaluation of numerous other mannosylated liposomal systems for potential MR-macrophage or dendritic cell binding were described elsewhere in the recent literature [129,130,131,132,133,134,135]. Alternatively, oil-in-water (O/W) emulsions were used as colloidal drug carriers for various therapeutic applications and as model particles for cell adhesion modeling [136,137,138]. The functionalized microparticles composed of a fluorescent mannolipid adsorbed on emulsion droplets were used to study the activation of MR-mediated phagocytosis in macrophages [139].

### 3.3. Mannosylated Nanoparticles (NPs)

Apart from liposomal systems, numerous other nano-constructs were investigated for their use in effective drug delivery to macrophages, including nanoparticles, carbon nanotubes, dendrimers, micelles, and polymeric particles. Nanoparticles can be prepared in various shapes and sizes, depending on various preparation methods and different starting materials. Different characteristics of nanoparticles, such as size, surface properties, and charge, can influence the interaction between nanoparticles and macrophages. For example, a smaller NP size (<100 nm) is preferred due to the slower removal from blood [140,141]. Moreover, NPs can be designed to specifically stimulate an immune response by interacting with immune components in blood, which causes easier recognition by macrophages [142].

Organic nanoparticles, particularly polymeric nano-objects and micelles, are often mentioned in the recent literature, considering that polymers reduce the often-limiting properties of nanomaterials, such as their low solubility, biocompatibility, and demanding preparation [143,144]. Glycopolymers can be synthesized either by post-polymerization conjugation or by the polymerization of glycosylated monomers. Modern polymerization methods can control the molecular weight, chemical functionality, and polymer architecture [145]. Even though the attachment of mannose onto preformed carriers through non-covalent conjugation includes the involvement of relatively weak nonspecific forces in the interaction between mannose and the carrier surface, which can result in the premature detachment of the ligand before its access to the target, the non-covalent fusion or coating of the previously prepared NPs with targeting ligands is also one of the well-known methods of preparing functional NPs [13,146]. For example, mannose decoration can be achieved via a self-assembly process, in which PEG-poly(propylene oxide)-PEG copolymer (F127-polymer) and tannic acid layers are physically crosslinked with the hydroxyl groups of mannose [147]. As in the case of the preparation of functional liposomes, the conjugation of mannosylated nanoparticles with PEG was used to provide higher stability, solubility, and immunogenicity. In addition, the PEGylation of mannose-modified NPs extended the circulation time and allowed for accumulation in the tumor site in studies related to tumor treatment. For example, Zhu et al. developed PEG-sheddable mannosylated NPs in order to target TAMs in the acidic tumor microenvironment. Recently, PEG-coated calcium zoledronate nanoparticles with conjugate mannose were designed [72]. Zoledronate (Zol) is a third generation of drugs known as bisphosphonates that displayed selective cytotoxicity to TAMs but a short half-life in cancer patients’ circulation [148]. The Zol-NPs specifically targeted TAMs via interaction with macrophage MR, which resulted in enhanced cellular internalization, tumor cell elimination, and restrained tumor growth. In addition, Chen and co-workers studied the optimal structural configuration of mannosylated PEG-conjugate type nanocarriers for targeting the MR on macrophage cells [149]. Their in vitro optimized parameters showed that a small PEG carrier and two mannose units per nanocarrier that are spaced 56 Å apart display the best targeting potential. In an additional study recently published, the optimal PEG and mannose-bearing nanocarriers were further modified for the application of copper-free click chemistry for precise attachment to the surface of a much larger, nanoscale macromolecular carrier [150]. The designed nanoparticles displayed selective targeting to anti-inflammatory rat peritoneal M2 macrophages. Several other studies explored mannosylated PEG-coated nanoparticle recognition via macrophage MR as a way to stimulate macrophages [151]. Since there is always a possibility that the flexible long PEG chains could shield the mannose structure and, thereby, prevent interaction with the MR, a stearoyl moiety was also introduced to overcome this structural problem [152,153].

Larger polymeric particles, with high functional group tolerance, can be easily prepared via reversible addition–fragmentation chain transfer (RAFT) polymerization and by using the recently reported upgraded version, called the automated synthesis of quasi-block copolymers via sequential RAFT polymerization [154]. Ortega et al. synthesized mannosylated lignin NPs to enhance the selective delivery of short nucleotide sequences to TAMs via MR [155]. These targeted NPs are biocompatible in vitro and in vivo at physiologically relevant doses, and thus, this study is the first to demonstrate mannose-conducted preferential mRNA delivery to TAMs in an in vivo model. Recently, Rushworth et al. prepared a series of fluorescently labeled RAFT polymers based on a mannose monomer scaffold that can be assembled into mannosylated NPs and successfully internalized into macrophages via a macrophage MR [156].

Apart from synthetic polymers, the biocompatible and biodegradable lignin polymer recently started to be used as a drug delivery system. Lignin nanoparticles (LNPs) were functionalized with the “mUNO” hexapeptide to target MR on TAMs, which resulted in a meaningful shift in the immune cells in the tumor microenvironment towards an anti-tumor immune state. In addition, the co-administration of the vinblastine with mUNO-LNPs enhanced its antitumor effect [157]. Also, mannosylated biodegradable chitosan nanoparticles were newly developed for macrophage remodeling in case of a fight with a Candida albicans infection. In this paper, mannose motif-enhanced macrophage targeting was emphasized [158]. Various other chitosan-based NPs for drug delivery were recently reported [159,160]. Mannose coating can enhance the uptake of the nanocarriers by alveolar macrophages in the spleen, liver, lymph nodes, and lungs [161,162]. More specifically, mannose significantly increased the uptake of gelatin NPs by alveolar macrophages for Mycobacterium tuberculosis treatment [163]. Gelatin is a natural macromolecule hydrolyzed from collagen, which is highly biocompatible and biodegradable under physiological conditions. When incorporated into mannosylated nano-carrier systems, gelatin is used to create targeted and controllable therapeutic-release carriers for various diseases [164,165,166,167]. Another FDA-approved poly lactic-co-glycolic acid (PLGA) that was a biocompatible and biodegradable polymer was functionalized with mannose, mannan, and mannosamine moieties using a carbodiimide reaction in order to prepare the nanoplatform for macrophage activation in Leishmaniasis therapy [168]. Recently, Zlotnikov et al. designed mannan-grafted cyclodextrin polymers, which showed a great capacity for the loading of antibacterial drugs and their adjuvants, as well as its delivery to macrophage MRs [169]. Within this work, it was presented that oligo- and polymer mannose conjugates grafted with cyclodextrins and polyethyleneimines possess a high loading capacity of 8–20% of the weight of the therapeutic substance and the ability to deliver it to macrophage MRs. In addition, flow cytometry studies showed a high-affinity absorption of the trimannosylated compound on macrophages (95.5%). Other mannose-functionalized NPs self-assembled from cyclodextrins were designed in order to facilitate the delivery of doxorubicin to breast cancer cells that overexpress the MR [170]. Furthermore, Bellato and co-workers pointed out the mannosylation of glycopolycations as an efficient strategy to target immune cells in cancer vaccination via MR [20].

In addition, polymeric micelles are self-assembled amphiphilic polymers with a hydrophobic tail and hydrophilic head where polymer concentrations are above critical micelle concentrations [171]. Depending on the hydrophobic and hydrophilic parts and solvents, micelles can take different shapes, including inverse micelles, spheres, tubules, mixed and cylindrical micelles, worm-like nanocrystal micelles, and so on [172]. In the last few decades, polymeric micelles were extensively used as novel drug vehicles, which can efficiently comprise and deliver various hydrophobic drug molecules to target tissues and subcellular organelles [171,173,174,175,176,177]. Like other nanoparticles, polymeric micelles can be functionalized with specific ligands to improve macrophage targeting efficiency. Recently, mannosylated mixed micelles were developed for the specific delivery of dasatinib (an approved drug for chronic myeloid leukemia) to TAMs, leading to their depletion and the suppression of tumor growth [178]. Also, mannosylated peptide-based micelles were synthesized, and their targeting potential for mannose-binding receptors was studied in order to design a suitable platform for immune modulation [179]. Additionally, mannose-coated polymeric micelles for targeted therapeutic siRNA delivery to human and murine macrophages were successfully synthesized [27]. Mannosylated micelles successfully delivered therapeutic mRNA and induced a shift in gene expression from an M2 phenotype toward an inflammatory M1 phenotype. Yin et al. developed mannosylated polymeric micelles from amphiphilic biodegradable polyesters, further modified with the lipophilic cancer drug doxorubicin. A mannose ligand was introduced via a highly efficient click chemistry protocol, and the authors emphasized that the mannose ligand was responsible for both the stabilization of micelles under physiological conditions and for the active targeting of cancer cells expressing MRs [180].

Mannosylated dendrimers are branched dendritic structures that can carry multiple and measurable mannose residues and also showed a significant macrophage uptake improvement [181]. The mannose-coated dendrimer-based antigen delivery system displayed several advantages in synthetic protocol and product characterization, as well as exceptional adjuvanticity in in vitro and in vivo models [182]. Polyamidoamine (PAMAM) dendrimers were widely applied due to their commercial availability, water solubility, and relatively high biocompatibility. To effectively target inflamed microglia cells, Sharma and co-workers presented the synthesis of mannose-attached, hydroxyl-terminated PAMAM dendrimers by applying a combination of the regioselective copper-catalyzed click reaction and esterification reaction [183]. Herein, the conjugation of mannose to the dendrimer system utilized MR-mediated endocytosis, as well as an in vivo brain uptake in inflammation-induced cerebral palsy rabbit model. Recently, another research related to the application of mannosylated PAMAM dendrimers as carrier molecules for imaging agents was reported [184]. Within the work, the authors emphasize their hypothesis that mannose residues on dendrimers can potentially be recognized by MRs, as well as by a variety of other endocytic molecules. In addition, He et al. synthesized mannose-functionalized dendrimeric PAMAM NPs for targeted delivery to atherosclerotic plaque-associated macrophages in in vivo studies [185].

## 4. Conclusions and Future Perspectives

MRs can be used for various applications, including the development of bioimaging tools and therapies for infectious diseases, cancer, and autoimmune disorders. Potential future perspectives for mannose-receptor targeting delivery involve a better understanding of receptor-binding patterns, resulting in more specific mannose receptor ligands. Mannose receptor targeting involves the delivery of agents directly to cells with mannose receptors expressed on their surface (pathogens in infectious diseases or certain cancer cells with overexpressed mannose receptors) and in the regulation of immune responses by modulating the activity of mannose receptors. The modulation of immune responses is important for infectious diseases and autoimmune responses but also for boosting the immune system against infections or cancer. The activation of certain immune responses can also be archived by the synergistic activation of mannose receptors as a CRL type of pattern recognition receptor and other classes such as TLR or NOD2 receptors. Targeting these receptors could be employed in the design of vaccines to enhance antigen uptake and presentation, leading to more effective immune responses. Additionally, mannose receptor-targeted drug delivery can be more precise through the use of mannose-functionalized delivery systems, which could improve the pharmacological properties of drugs and reduce side effects. The major advantages of mannose-based carrier systems are listed in Table 1. Further research is needed to overcome limitations for the broader application of mannose glycomimetics in mannose receptor targeting related to their preparation (demanding synthesis, purification, and scale-up, especially for highly functionalized NPs and branched glyco-structures) and their stability in physiological conditions (blood and tissues).

## Figures and Tables

**Table 1:** The major advantages of mannose-based glycomimetics as ligands for MR.

Mannosylated Carrier System	Advantages
Antigens/Proteins/Peptides	Significant enhancement of the cellular and humoral immune response through APC activation and antigen uptake
	Increased peptide solubility and oral bioavailability
	Increased specificity of peptide vaccine candidates toward the desired cellular target
Lipids and liposomes	Increased cellular uptake
	Promoted cross-presentation of model antigen
	Induction of strong antitumor effects in tumor-bearing mice
	Improved cytosolic delivery of therapeutics to specific macrophages
Nanoparticles	Improved pharmacokinetic profile
	Effective internalization in targeted cells
	High binding affinity for targeted cells, successful delivery of drugs, and therapeutic mRNA

## Data Availability

Data are contained within the article.
